# Recommendations on Rapid Diagnostic Point-of-care Molecular Tests for Respiratory Infections in the United Arab Emirates

**DOI:** 10.2174/0118743064319029240815074449

**Published:** 2024-12-17

**Authors:** Liliane Dhaini, Rashi Verma, Mazin A Gadir, Harmandeep Singh, Mohamed Farghaly, Tamir Abdelmutalib, Amar Osman, Khulood Alsayegh, Somaia Bin Gharib, Bassam Mahboub, Eldaw Suliman, Sofia Konstantinopoulou, Srinivasa Rao Polumuru, Sandeep Pargi

**Affiliations:** 1 Consulting and Analytics, IQVIA, Dubai, United Arab Emirates; 2 Consulting and Analytics, IQVIA, Bengaluru, India; 3 Strategic Partnership, IQVIA, Dubai, United Arab Emirates; 4 Engagement Management, IQVIA, Dubai, United Arab Emirates; 5 Family Medicine Department, Dubai Health Insurance Corporation, Dubai, United Arab Emirates; 6 Medical Practices Ethics-Healthcare Workforce Planning Division, DOH Healthcare Workforce Sector, Department of Health, Abu Dhabi, United Arab Emirates; 7 Policy Advisement, Dubai Health Authority, Dubai, United Arab Emirates; 8 Family Medicine Department, Dubai Health Authority, Dubai, United Arab Emirates; 9 Clinical Standards and Guidelines, Dubai Health Authority, Dubai, United Arab Emirates; 10 Pulmonary Medicine Unit, Dubai Health Authority, Dubai, United Arab Emirates; 11 Rashid Hospital, Dubai, United Arab Emirates; 12 Health Research and Policies, Dubai Health Authority, Dubai, United Arab Emirates; 13 Pulmonology and Sleep Medicine Departments, Sheikh Khalifa Medical City, Abu Dhabi, United Arab Emirates; 14 Internal Medicine Department, NMC specialty hospital, Al Nahda, Dubai, United Arab Emirates; 15 Pulmonology Department, Prime Medical Hospital, Dubai, United Arab Emirates

**Keywords:** COVID-19, Respiratory syncytial viruses, Respiratory tract infections, Influenza, Point-of-care testing, Group A streptococcus

## Abstract

Traditional testing methods in the Middle East Region, including the United Arab Emirates (UAE), particularly the testing of Respiratory Syncytial Virus (RSV), influenza, group A streptococcus (GAS), and COVID-19 have the potential to be upgraded to new and advanced diagnostics methods that improve lead time to diagnosis, consumption of healthcare resources and patient experience. In addition, based on the research, it was reported that there is an underreporting of respiratory cases, overuse of antibiotics, and prolonged hospitalizations which is posing pressure on UAE healthcare stakeholders. A literature review was done exploring UAE's current diagnostic practices, recommended guidelines, diagnostic gaps, and challenges in RSV, GAS, Influenza, and COVID-19. This was followed by stakeholder discussions focusing on assessing current diagnostic practices, usage of rapid molecular point-of-care (POC) diagnostic tests, current gaps in diagnosis, targeted profiles for POC testing, and potential impact on patient management for targeted respiratory infections. A round table discussion with healthcare experts, insurance experts, key opinion leaders, and pulmonologists discussed challenges and opportunities in treating respiratory diseases. UAE healthcare stakeholders suggest that introducing alternative and up-to-date diagnostic methods such as POC molecular testing is expected to improve healthcare outcomes, optimize resources, and develop a robust case management of respiratory tract infections. It is essential to emphasize that by introducing POC testing, precision medicine is reinforced, efficiency is achieved, and the overall management of population health is enhanced.

## 1 INTRODUCTION

Respiratory tract infections (RTIs), including viral and bacterial, are a profound concern to global health, particularly due to their ubiquitous nature with alarming rates of morbidity and mortality [1]. As of 2019, these infections have been recognized as the fourth predominant cause of death, with 2,603,913 fatalities reported globally [1-5]. In the United Arab Emirates (UAE), the prevalence of Respiratory Syncytial Virus (RSV) in Lower respiratory tract infections (LRTIs) is 28.6% among young children and infants, becoming the most common cause of bronchiolitis (51%) [6-8]. Worldwide, these annual epidemics of seasonal influenza are estimated to result in 3 to 5 million cases of severe illness and reach 650,000 respiratory deaths. Influenza in the Middle East and North Africa (MENA) region has an overall prevalence of 23.3% and in UAE, it contributes to 20% of all RTIs. Moreover, the prevalence of Group A Streptococcus (GAS) in the MENA region ranged from 2.5% up to 42.4% in pharyngitis patients and from 2.4% up to 35.4% in healthy carriers [9]. In October 2022, doctors in the UAE alarmed residents about a “tripledemic”, one that included the flu, RSV, and COVID-19, being on the rise [10]. Even though COVID-19 cases were declining in the UAE, more school-age children were falling sick, with cases of flu and RSV infections increasing [11].

Diseases such as influenza, RSV, GAS, and COVID-19 manifest with many overlapping symptoms [12-14], making clinical differentiation a challenging task. Conven- tional diagnostic methodologies, with their prolonged turnaround times(TATs), often culminate in delayed therapeutic interventions, extended hospitalizations, and an augmented risk of complications [15]. This jeopardizes patient health and imposes a substantial economic burden on healthcare systems [15-28].

The absence of molecular point-of-care tests (POCTs) for respiratory diseases in the UAE is a concern raised by the healthcare community during a roundtable discussion. UAE Key opinion leaders (KOLs) highlighted a lack of local literature and evidence-based data on the utilization of these tools within local clinical settings and that a real opportunity exists to advance the diagnosis of respiratory cases [29]. As adopted globally, rapid POCTs have proved to support medical professionals in achieving a timely and accurate diagnosis at the patient’s bedside within various clinical settings, thus preventing rapid disease spread and outbreaks. The potential repercussions of not integrating POCTs into the healthcare framework include treatment lags, augmented patient distress, escalated mortality indices, and heightened economic strain on the healthcare system [17, 18 ,30].

This paper aims to describe the benefits of rapid molecular POCTs for respiratory infections by defining their role in enhancing patient outcomes, supporting public health, reinforcing efficiency and economic viability, and their impact on the healthcare system in the UAE.

## 2 REVIEW PROCESS

This publication is based on a research study that was conducted by IQVIA^1^. This included feedback and interviews with multiple stakeholders in the UAE, including providers, caregivers, payers, and regulators. The key findings and recommendations were developed based on a detailed literature review and individual discussions with KOLs followed by a workshop.

A thorough literature review was conducted around the disease epidemiology, investigating the current diagnostic practices, recommending international and national guidelines that are adopted within UAE, diagnostic gaps, and challenges in the diagnosis of RSV, Streptococcus group A, influenza, and SARS-CoV-2. In addition, in-depth discussions with multiple stakeholders, including experts in policy making, payors, and KOLs were established. These discussions focused on assessing the current diagnostic practices, usage of POC, rapid diagnostic tests, especially molecular, current gaps in diagnosis, targeted profiles for point-of-care testing, and potential impact on patient management for targeted respiratory infections.

Further feedback and a brainstorming session were led during a round table discussion with experts in healthcare policies, insurance, KOLs, and pulmonologists, where challenges and opportunities in treating respiratory diseases were discussed. As a result, a publication was developed. Stakeholders reviewed literature evidence up to August 2023, as well as their own clinical and personal expertise when high-quality evidence was lacking. If there were any disagreements, the coordinators' criteria were used to decide. Additionally, an analysis of real-world examples where POC rapid tests have been implemented and their outcomes in the UAE were discussed and included based on the discussion.

^1^About IQVIA: IQVIA (NYSE:IQV) is a leading global provider of advanced analytics, technology solutions, and clinical research services to the life sciences industry. IQVIA creates intelligent connections to deliver powerful insights with speed and agility — enabling customers to accelerate the clinical development and commercialization of innovative medical treatments that improve patient healthcare outcomes. With approximately 86,000 employees, IQVIA conducts operations in more than 100 countries. Learn more at www.iqvia.com.

### 2.1 Disease Burden of Common Causative Organisms of Respiratory Infections

#### 2.1.1 Influenza A and B

Influenza A and B are highly prevalent worldwide and in the UAE [31-35]. Individuals aged 65 years or older have a higher risk of experiencing severe influenza symptoms and developing serious complications [36]. In the MENA region, Influenza is reported to have an overall prevalence of 23.3% while Influenza A contributes to 67.9%. Within UAE, Influenza contributes to 20% of total RTIs, with approximately 466 per 100,000 deaths reported in Dubai in 2019.

#### 2.1.2 Respiratory Syncytial Virus (RSV)

Respiratory syncytial virus (RSV) is a frequent cause of LRTI in infants and young children, especially those less than 2 years old. Recently, RSV has also become a common pathogen among elderly and immuno- compromised patients [37]. It has an estimated global incidence of over 30 million cases in children ≤ 5 years [37]. As infants are not well protected by maternal immunoglobulins during their first year of life, about 80% of RTIs in children <1 year are caused by RSV [37,38].

Within MENA, RSV has an overall prevalence of 24.24% for RTIs, while in the UAE, it reaches 28.6% in LRTIs among children under 6 and infants below 2 years old (higher prevalence than the overall MENA region). However, there is scarce data regarding the epidemiology of RSV in MENA and the UAE [6-8].

#### 2.1.3 Group A Streptococcus (GAS) Infections

Group A streptococcus (GAS) infections cover a broad spectrum of conditions with various clinical presentations. Repeated GAS infections can also cause autoimmune complications, including acute rheumatic fever (ARF), resulting in rheumatic heart disease (RHD) [39,40]. It is estimated that GAS causes 20% to 40% of cases of pharyngitis in children [41,42]. It is considered a major contributor to the global mortality rate from infectious diseases, resulting in approximately 500,000 deaths annually [43]. Estimating the global burden of Strep A disease is a challenging task due to the variety and complexity of the associated disease spectrum [44].

In the MENA region, the prevalence of GAS ranged from 2.5% up to 42.4% in pharyngitis patients and from 2.4% up to 35.4% in healthy carriers [9]. Since the symptoms of GAS and viral pharyngitis overlap, clinical scoring systems are not always accurate enough to make a diagnosis [11]. Therefore, most global guidelines recommend using microbiological cultures to diagnose GAS [45].

#### 2.1.4 For COVID-19

For COVID-19, there have been about 700 million reported cases and seven million deaths worldwide [46]. In the UAE, the first case of COVID-19 was identified on 29 January 2020 [47], and over one million cases and two thousand deaths were reported till now [46]. The gold standard for diagnosing COVID-19 is still RT-PCR. Nevertheless, new molecular techniques and immunoassays have emerged.

### 2.2 Current State of Diagnosis - Urgent Need for Rapid Molecular POC Tests

According to the stakeholders in the public and private sector in UAE, the diagnosis of respiratory infections in the UAE is mainly conducted through clinical assessment of symptom and presentation. Mainly severe cases are tested through conventional laboratory tests, principally RT-PCR and multiplex panel tests [29]. The usage of rapid tests, such as the lateral flow diagnostic (LFD) antigen tests for COVID-19 and influenza in emergency rooms is limited in both the public and private sectors. While samples are collected at POC, the tests are performed in the lab, which dilutes the purpose of immediate test results, being the key benefit of rapid tests [29].

Ultimately, conventional laboratory and diagnostic methods present several limitations that adversely impact patient care. There is an overlap of symptoms and seasonality. Both adult and pediatric patients who are infected with diseases such as influenza, RSV, GAS, or COVID-19 often manifest a broad spectrum of similar symptoms—ranging from fever and cough to sore throat, fatigue, headache, and gastrointestinal symptoms [12-14] (Table **1**). This overlap, in addition to the seasonal concurrence of diseases like RSV and influenza, complicates the clinical diagnosis and impacts the proper medical management [19].

An additional prominent issue includes the extended TAT, averaging between 48 and 72 hours [15,29]. This delay hinders timely hospital admissions, prolongs hospital stay, complicates diagnosis and management, and delays isolation measures implementation [29]. In emergency scenarios, the reliance on lab-based testing can result in significant diagnostic delays, amplifying the risk of severe complications. It has been demonstrated that there is an extra patient death in 82 hospital admissions whose time to inpatient bed transfer is delayed beyond 6-8 hours from admission [48].

Another challenge is the inability of current tests to discriminate between pathogens causing active disease versus those colonizing asymptomatic carriers [20]. This is further exacerbated by the suboptimal sensitivity and specificity of antigen and culture-based methods compared to molecular techniques [15]. The lack of rapid, accurate, and standardized tests often leads to the inappropriate use of empiric antibiotics, thereby delaying effective treatment and contributing to the rise of antimicrobial resistance [15-20].

The challenge of low sensitivity is distressing where over 25% of positive Strep A cases are missed due to the low sensitivity of the antigen rapid test. Additionally, RSV antigen testing achieves high sensitivity only in children, thus methods that offer high sensitivity in all patient groups are better to use [49].

Moreover, for high-risk populations—including the elderly, immunocompromised patients, pregnant women, individuals with co-morbidity or coinfection, and young children or premature infants—the performance of conventional microbiology is notably inadequate [21-28]. In addition, healthcare systems continue to face large costs and high resource utilization due to Flu, RSV, and COVID-19 with an average public expenditure per hospitalized patient of ~$4,578 [50], $5,325 [51], and $935 [52] respectively. Notably, Strep A pharyngitis and its sequelae bring a considerable economic burden to healthcare systems globally of $205 per case [53].

Based on the literature as well as inputs from multiple stakeholders (regulators, KOLs, and payors), there are multiple barriers which are limiting the POCT adoption, further enhancing the diagnostic gaps. These include the need for guidelines enforcement for POCTs since there are recent recommendations for the usage of rapid POCTs under the Ejadah^2^ program for Flu and Strep A [29,54], however, it requires further reinforcement. Additionally, there is also limited awareness among payors on the long-term impact of rapid POC molecular tests restricting their implementation. Other barriers include the limited interest of labs towards rapid POCT, lack of awareness among the stakeholders on the ease of required training, and reduced awareness of the scientific evidence published around the benefits and usage of POCTs.

^2^Ejadah Program is launched by DHA and its insurance regulatory arm, Dubai Health Insurance Corporation (DHIC). This program is a digital-led programme focusing on value-based healthcare adoption in UAE. The programme aims to enhance oversight over the clinical, economic, and human-centric outcomes. Under the Ejadah program, multiple indications / disease areas have been identified, various KPIs / recommen-dations have been published in phased approach.

### 2.3 Benefits and Added Value of POCTs

#### 2.3.1 Time to Result

**Fig. (1):**
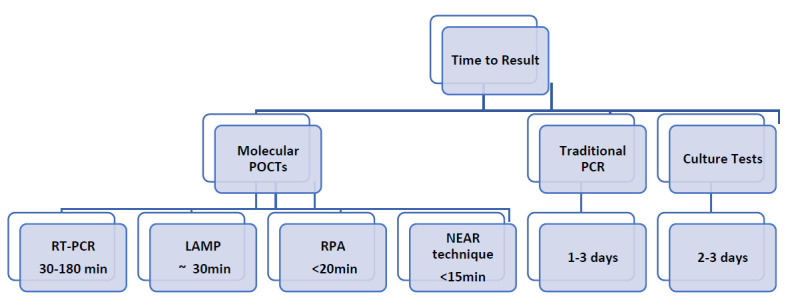
Time to results of POCTs versus traditional testing.

Molecular POCTs demonstrate distinct advantages over their non-molecular counterparts. While enzyme-linked immunosorbent assay (ELISA) and lateral flow immunoassays (LFA) have their merits, molecular POCTs— based on nucleic acid amplification technique (NAATs) [55], recombinase polymerase amplification (RPA), and loop-mediated isothermal amplification (LAMP), Iso- thermal Molecular Nicking Enzyme Amplification Reaction (NEAR)—are demonstrably superior to terms of both sensitivity and specificity [56,57]. The time to result demonstrated by such molecular techniques is highly beneficial, as seen with RPA producing results in less than 20 minutes [58], LAMP test within 30 minutes to an hour [59], and RT-PCR with only 30 minutes [60] (Fig. **1**). The latest NEAR technique-based assays can generate test results in 15 minutes [61], with tests for Strep A in less than 6 minutes, and have sensitivity and specificity levels of 96.6% and 94.4-96.1%. Thus, they parallel the high specificity and sensitivity of lab-based RT-PCR tests, all while offering cost-effectiveness [55-57]. They have additionally been tested in multiple studies [61-63] compared to traditional methods, where they demons- trated the smallest inefficiency rate (0.5%) and a much shorter time to produce positive results. When compared to the SARS-CoV-2 detection platforms from other competitor brands, NEAR-based assay currently offers the fastest detection time [61,64]. By far, this new technology is a better method of choice for use as a gold standard test at point-of-care [65-67].

#### 2.3.2 Clinical Effectiveness

POCTs have been associated with a reduction in antibiotic prescriptions and ancillary investigations, thereby expediting patient discharges [68,69]. This acceleration in patient care has a direct impact on hospital admissions (reduction rates ranging between 9.8% to 14%) [70,71], medical procedures, and the overall financial burden on healthcare systems [70,72-78]. According to an insurance industry expert, it was found that 86% of the patients are prescribed antibiotics on the first visit. There is an estimated cost of around 665 billion dirhams (~181 billion US dollars) over 2 years for unjustified use of antibiotics [29]. These rapid diagnostics also play a pivotal role in mitigating the transmission of hospital-acquired influenza infections and the timely initiation of appropriate isolation protocols [70,72-78].

Moreover, intensive care unit (ICU) admissions were higher in the standard PCR group than in the rapid PCR group of respiratory viruses. Over 80% of patients were subsequently discharged home, and in-hospital deaths occurred in 3.6 to 7.7% of rapid PCR group patients. The overall median TAT was significantly shorter for rapid PCR compared to standard multiplex PCR (2.3 h versus 27.4 h, P < 0.01) [79].

Additionally, the use of a rapid influenza and RSV molecular test improves the clinical management of patients admitted to the emergency department by providing fast and reliable results. Their additional cost compared to antigen tests should be balanced against the benefit of their analytical performance, leading to efficient reductions in the need for isolation and antibiotic use [80].

#### 2.3.3 Cost-effectiveness

Multiple studies implicated significant cost savings with the usage of rapid molecular POCTs. In the National Health Service (NHS) medical assessment units and emergency department, there was an estimated saving of around £242,730 per 1000 adults presenting with influenza-like symptoms to the usage of rapid molecular POCT [75]. An Irish study demonstrated that patients who had a rapid molecular POCT influenza test cost 67% less than those who did not [81]. Similarly, a study on Strep A complications found cost reduction by 11% after POCT, while Long-COVID had an avoidable cost of $17,500 per patient [82,83]. Furthermore, empirical studies indicate a 26% and 2.5% reduction in the cost of illness for elderly individuals and adults with influenza-like symptoms, respectively [84]. This cost-effectiveness is attributed to a decrease in hospitalizations, fewer follow-up visits, and a reduced need for additional diagnostics and antibiotics [85]. A 50% reduction in hospitalization cost was observed when testing flu-like illnesses using a rapid molecular POCT (NEAR technique assay) compared to clinical judgement, with cost savings of antibiotics reaching 40% [84].

To sum up, several molecular POCTs enhance the efficiency of healthcare delivery [86] with available tests that can identify multiple respiratory pathogens from a single patient specimen through a single test run with ease-of-use testing requiring minimum training.

### 2.4 Stratified Implementation of Rapid Molecular POCTs: Implications for Patient Profiles and Healthcare Settings in the UAE

**Fig. (2):**
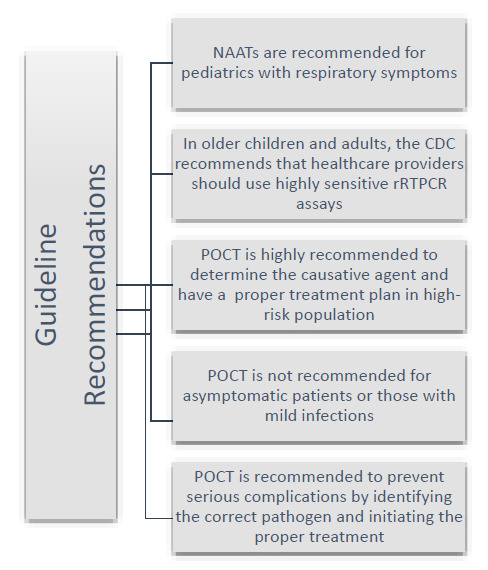
Summary of guidelines’ recommendations.

The utilization of rapid molecular POCTs is strongly advocated across a diversity of patient demographics. Endorsed by multiple authoritative guidelines such as the Infectious Diseases Society of America, the American Society for Microbiology, and the French Haute Autorité de santé, these tests are indispensable for symptomatic patients [87] (Fig. **2**).

For hospitalized patients, the application of POCTs is congruent with American clinical guidelines and is anticipated to yield analogous efficacious outcomes in the UAE, given the parallelism in the prevalence of respiratory diseases with global statistics [87,88]. For populations with compromised immune systems and pediatric cohorts—particularly those with congenital diseases or immunodeficiency—empirical evidence substantiates the clinical advantages of POCTs including the amelioration of acute medical conditions and the mitigation of hospitalization expenditures [89-91]. In geriatric patients and those afflicted with chronic medical conditions, the utilization of POCTs is advocated to attenuate the risk of adverse clinical outcomes and mortality [87, 92, 93].

Within the emergency departments of the UAE, the prevailing diagnostic paradigm involves laboratory molecular assays, producing additional financial burdens and temporal delays, with TATs oscillating between 6 to 24 hours [88]. A plethora of studies, inclusive of systematic reviews, corroborate the efficacy of POCTs in curtailing supplementary diagnostic evaluations and the prescription of antibiotics in primary healthcare centers (PHCs) and ambulatory care settings, with a pronounced impact observed in pediatric emergency departments [94]. This is also important in special treatment centers such as outpatient dialysis centers where patients are treated at least three times per week, thus repeated exposure to health care staff and all patients treated at the same time in a single session might lead to an excessive spread of respiratory diseases [95].

In inpatient and ICU settings, the successful deployment of POCTs has been documented to curtail disease transmission rates and enhance patient outcomes [96,97]. Moreover, their recommendations extend to all patients necessitating hospitalization due to acute respiratory ailments and those experiencing acute exacerbations of chronic cardiopulmonary conditions [87]. The Dubai Health Insurance Corporation's publication [54] advocating the use of POCT underscores the imperative for its expansive implementation across the UAE. These recommendations include the usage of POC testing for influenza in outpatient settings, rapid influenza molecular assays in inpatient settings, and testing of GAS pharyngitis using rapid antigen tests and NAATs [29].

### 2.5 Recommendations for Successful Implementa-tion of POCTs

With the evident benefits of rapid POCT for respiratory infections, a roundtable discussion that gathered stake-holders from the UAE healthcare system synthesized the following recommendations:

#### 2.5.1 Reinforcement of Clinical Guidelines

Different regulatory bodies, including the Ministry of Health and Prevention, the Department of Health - Abu Dhabi, and the Dubai Health Authority (DHA), among others, are advised to announce clinical guidelines with recommendations regarding rapid POCTs related to respiratory infections and the reinforcement of their adoption.

#### 2.5.2 Collaboration among the UAE Healthcare Community

There is a call directed to private and public insurance companies to support the adoption of clinical guidelines and update policy coverage where applicable. POC molecular rapid tests generate savings, with up to 50% reduction in the cost of isolation and a 100% reduction in secondary transmission cost due to quick turnaround time [98].

#### 2.5.3 Generation of Evidence-based Local Data

A collaboration among the UAE healthcare community, presented in hospitals and clinical settings under the Emirati government, is key to conducting local clinical studies that support the advancement in diagnostic testing.

#### 2.5.4 Enhance Awareness

Awareness of the latest portable devices, minimum resource requirements, and training to healthcare professionals is needed to further develop [84].

A strong action plan is required, including implementing the aforementioned recommendations promptly which can be executed in a phased approach over the next 3-5 years, with regular monitoring and feedback evaluation from all associated members of the healthcare system to ensure the desired outcomes are achieved. This call to action for stakeholders can ultimately lead to a more robust, equitable, and responsive healthcare system to the needs of the UAE population.

## CONCLUSION

In light of the evidence presented, it is evident that there are opportunities for improving the diagnosis of RTIs, as advised by the healthcare stakeholders in the UAE. The limitations of certain traditional diagnostic methodologies, coupled with the overlapping symptomatology of various RTIs, present a need to shift the diagnostic approach. Rapid Molecular POCTs have emerged as a scientifically validated solution, offering timely and accurate results, which are paramount for optimal patient management. Adoption of advanced techniques has an impact on cost savings, reduced hospitalizations, and optimized resource utilization.

## AUTHORS’ CONTRIBUTION

It is hereby acknowledged that all authors have accepted responsibility for the manuscript's content and consented to its submission. They have meticulously reviewed all results and unanimously approved the final version of the manuscript.

## Figures and Tables

**Table 1:** Common overlapping symptoms among RSV, SARS-CoV-2, Group A streptococcus, and influenza infections.

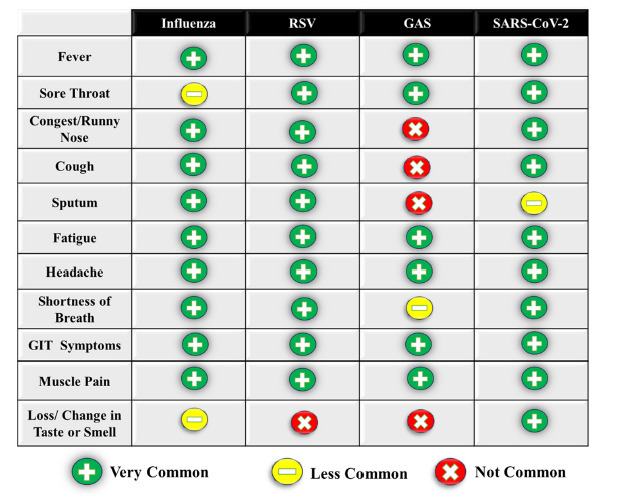

## LIST OF ABBREVIATIONS

UAE: = United Arab Emirates
RSV: = Respiratory Syncytial Virus
GAS: = Group A streptococcus
POC: = Point of Care
RTIs: = Respiratory tract infections
LRTIs: = Lower respiratory tract infections
MENA: = Middle East and North Africa
TAT: = Turnaround time
POCT: = Point-of-care test
KOLs: = Key opinion leaders
ARF: = Acute rheumatic fever
RHD: = Rheumatic heart disease
LFD: = Lateral flow diagnostic
ELISA: = Enzyme-linked immunosorbent assays
LFA: = Lateral flow immunoassay
NAATs: = Nucleic acid amplification techniques
RPA: = Recombinase polymerase amplification
LAMP: = Loop-mediated isothermal amplification
NEAR: = Nicking Enzyme Amplification Reaction
DHIC: = Dubai Health Insurance Corporation
ICU: = Intensive care unit
